# Longitudinal humoral immune response and maternal immunity in horses after a single live-attenuated vaccination against African horse sickness during the disease outbreak in Thailand

**DOI:** 10.14202/vetworld.2023.1690-1694

**Published:** 2023-08-19

**Authors:** Nutnaree Kunanusont, Machimaporn Taesuji, Usakorn Kulthonggate, Khate Rattanamas, Thanongsak Mamom, Kosin Thongsri, Thawijit Phannithi, Sakchai Ruenphet

**Affiliations:** 1 Clinic for Horse, Faculty of Veterinary Medicine, Mahanakorn University of Technology, 140 Cheum-Sampan Rd. Nong Chock, Bangkok 10530 Thailand; 2 Department of Immunology and Virology, Faculty of Veterinary Medicine, Mahanakorn University of Technology, 140 Cheum-Sampan Rd. Nong Chock, Bangkok 10530 Thailand; 3 Department of Pathology, Faculty of Veterinary Medicine, Mahanakorn University of Technology, 140 Cheum-Sampan Rd. Nong Chock, Bangkok 10530 Thailand; 4 Department of Veterinary and Remount, Division of First Livestock and Agriculture, The Veterinary Hospital, Royal Thai Army, 57 Koh Samrong Subdistrict, Mueang District, Kanchanaburi Province 71000 Thailand

**Keywords:** African horse sickness, antibody, maternal immunity, stage of gestation, vaccination

## Abstract

**Background and Aim:**

African horse sickness (AHS) has become a newly emerging disease after an outbreak in northeastern Thailand in March 2020. Mass vaccination in horses with live-attenuated AHS virus (AHSV) vaccine is essential for AHS control and prevention. This study aimed to monitor the longitudinal humoral immune response before and after a single vaccination using a live-attenuated vaccine against AHS in stallions, mares, and pregnant mares, including maternal immunity in foals born from pregnant mares during the outbreak in Thailand.

**Materials and Methods:**

A total of 13 stallions and 23 non-pregnant and 21 pregnant mares were vaccinated with live-attenuated AHSV vaccines. Serum samples from selected horses were collected on the day of vaccination and 1, 6, 8, 9, 10, and 12-months post-vaccination. Furthermore, seven serum samples of foals born from vaccinated pregnant mares were collected on parturition date and 1, 3, and 6-months old. The antibody titer against AHS in all collected serum samples was evaluated using a commercial enzyme-linked immunosorbent assay kit. All data were analyzed for mean and standard deviation for each group of samples using a spreadsheet program. Antibody titers between times were analyzed using a one-way analysis of variance as repeated measurement, and antibody titers between horse groups were analyzed using a general linear model for statistically significant differences when p < 0.05.

**Results:**

In stallion and non-pregnant mare groups, there were no statistically significant differences in antibody titers in all 6 time periods after vaccination. The antibody titer in the pregnant mare group showed a non-statistically significant difference between each gestation stage, except at 8 months post-vaccination. Furthermore, increasing antibody titers on days 1 and 3 after receiving colostrum in foals indicate the major role of transcolostral antibody transfer for AHS.

**Conclusion:**

This study demonstrated that a single AHS vaccination using a live-attenuated vaccine could stimulate high antibody titers sufficient for AHS control and prevention during the outbreak in Thailand. Similarly, the antibody response of vaccinated horses of both genders, including various stages of pregnant mares, was statistically not different.

## Introduction

African horse sickness (AHS) is an infectious but non-contagious viral disease with high mortality and affects all Equidae species. This disease was included in the World Organization for Animal Health (OIE). African horse sickness is caused by the AHS virus (AHSV), an RNA virus of the family Reoviridae, genus Orbivirus. The AHSV is classified into nine serotypes based on viral capsid protein (VP)-2 [1, 2]. In Africa, AHS is classified as an endemic disease, an important health and trade-sensitive disease of equids [3]. Nevertheless, the disease has become a newly emerging disease after an outbreak in northeastern Thailand in March 2020 [4–6], during which AHSV serotype 1 was confirmed [5, 7]. The economic loss from an AHS outbreak is not only due to increased horse mortality rate but also due to movement restrictions, culling of infected horses, attempts to control *Culicoides*, and horse vaccination strategies [8].

Movement restrictions, quarantine of suspected horses, including other equine species, disinfection, and vector elimination, especially *Culicoides* biting midges, were applied during the AHS outbreak in Thailand according to the OIE regulation. At least 99 species of *Culicoides* have been reported in Thailand [9]. Nonetheless, mass vaccination in horses with a live-attenuated AHSV vaccine is required to control and prevent AHS [4, 7].

Therefore, this study was conducted to monitor the longitudinal humoral immune response before and after a single vaccination using a live-attenuated virus vaccine against AHS in stallions and non-pregnant and pregnant mares, as well as to monitor the maternal immunity in foals during AHS outbreaks in Thailand.

## Materials and Methods

### Ethical approval

Guidelines used for the care and use of animals were approved by the Animal Research Ethics Committee, Faculty of Veterinary Medicine, Mahanakorn University of Technology, Thailand, approval number ACUC-MUT-2021/003.

### Study period and location

This study was conducted from May 2020 to October 2022 in the biosecurity level-2 facilities of Virology and Molecular Diagnostic Laboratory, Faculty of Veterinary Medicine, Mahanakorn University of Technology, Bangkok, Thailand.

### Horses and sample preparation

A total of 13 stallions and 23 non-pregnant and 21 pregnant mares were vaccinated with a live-attenuated AHS vaccine (Onderstepoort Biological Products SOC Ltd., Co. South Africa). Serum samples from selected horses were collected on the day of vaccination and 1, 6, 8, 9, 10 and 12 months post-vaccination. Moreover, serum samples of foals delivered from vaccinated pregnant mares were collected on parturition date and 1, 3, and 6- months of age. All serum samples were stored at −30°C before testing.

### Antibody evaluation

The AHS antibody titer was evaluated using a commercial enzyme-linked immunosorbent assay (ELISA) kit, namely, INGEZIM AHSV COMPAC PLUS^®^ (INGENASA, Madrid, Spain). This kit is based on blocking ELISA, which reacts between the recombinant VP7 protein adsorbed on the ELISA plate and a peroxidase-conjugated AHS-VP7-specific monoclonal antibody. The blocking percentage (BP) was used to determine the antibody titer in this study, according to Taesuji *et al*. [10].

### Statistical analysis

All data were analyzed for mean and standard deviation for each group of samples using a spreadsheet program (Excel 2000; Microsoft Corporation, New Mexico, USA). Antibody titers between times were analyzed using a one-way analysis of variance as repeated measurement, and antibody titers between horse groups were analyzed using a general linear model for statistically significant differences when p < 0.05.

## Results

Table-1 shows the mean and standard deviation of BP before and after AHS vaccination in stallion and mare groups. On the vaccination day (day 0), both groups showed a negative result of antibody detection (BP < 45). An increasing antibody titer was observed (BP > 50) in all samples of the 6 time periods after vaccination with no statistically significant differences (p < 0.05) when compared between stallion and mare groups. However, there was a difference in antibody titers when samples from both groups were compared with samples collected on the vaccinated day (day 0).

**Table-1:** Blocking percentage (mean and standard deviation) of immune response before and after vaccination against African horse sickness in stallions and mares using blocking ELISA.

Groups	Vaccinated day	1 MPV	6 MPV	8 MPV	9 MPV	10 MPV	12 MPV
Stallion	23.11 ± 1.46^D^	92.91 ± 7.78^C^	109.70 ± 1.75^AB^	108.01 ± 3.05^A^	110.20 ± 1.84^B^	106.46 ± 4.00^A^	105.21 ± 4.02^A^
Mare	20.72 ± 7.55^C^	93.69 ± 9.33^B^	107.21 ± 3.87^A^	108.89 ± 3.60^A^	109.86 ± 1.68^A^	107.15 ± 4.63^A^	106.87 ± 2.27^A^
Stallion + mare	20.92 ± 7.25^C^	93.41 ± 8.69^B^	108.14 ± 3.44^A^	108.57 ± 3.39^A^	110.00 ± 1.72^A^	106.90 ± 4.37^A^	106.27 ± 3.07^A^

^A,B,C,^DValues within the same row with different superscripts mean statistically significant difference (p < 0.05), MPV=Month post-vaccination, ELISA=Enzyme-linked immunosorbent assay

In the pregnant mare group, there was a statistically significant difference in antibody titers in all stages of gestation after vaccination compared with antibody titers on a vaccinated day (day 0). Nevertheless, the antibody titers almost showed a non-statistically significant difference in each gestation stage after vaccination, except at 8 months post-vaccination (Table-2).

**Table-2:** Blocking percentage (mean and standard deviation) of immune response before and after vaccination against African horse sickness in pregnant mares using blocking ELISA.

Gestation stage	Vaccinated day	1 MPV	6 MPV	8 MPV	9 MPV	10 MPV	12 MPV
<3 month	19.25 ± 5.52^C^	95.14 ± 7.78^B^	107.31 ± 4.57^A^	109.43 ± 1.62^A^,^ab^	110.00 ± 1.61^A^	108.19 ± 2.95^A^	106.96 ± 2.74^A^
3–7 month	27.54 ± 5.58^C^	86.98 ± 13.16^B^	105.64 ± 4.66^A^	105.37 ± 6.11^A^,^b^	109.70 ± 0.00^A^	105.46 ± 9.09^A^	104.54 ± 5.23^A^
>7 month	16.84 ± 10.64^C^	94.17 ± 7.72^B^	107.72 ± 1.91^A^	110.26 ± 1.83^A^,^a^	110.15 ± 2.47^A^	107.32 ± 2.26^A^	106.45 ± 2.70^A^
All stages	20.72 ± 7.88^C^	92.97 ± 9.42^B^	107.00 ± 3.99^A^	108.66 ± 3.62^A^,^ab^	110.02 ± 1.75^A^	107.33 ± 4.82^A^	106.26 ± 3.42^A^

^A,B,C,D^Different superscripts in a row mean statistically significant difference (p < 0.05), ^a,b^Different superscripts in column mean statistically significant difference (p<0.05), MPV=Month post-vaccination, ELISA=Enzyme-linked immunosorbent assay

The BP in the foal group born from vaccinated pregnant mares is presented in Table-3. Four foals with low antibody titers on parturition day (before colostrum intake) showed high antibody titers at 1 month old after colostrum administration. Another 3 foals, after 24 h of colostrum administration, showed high antibody titers on parturition day, which remained detectable until 1 to 3 months of age after colostrum administration. The last 3 foals, with unknown passive antibody titers, showed an enhanced antibody titer response after vaccination at 6 months old.

**Table-3:** Blocking percentage of maternal immunity in foals before and after colostrum receiving.

Foal	Titer from vaccinated mare	Parturition day	1 month old	3 month old	6 month old	9 month old	10 month old	12 month old
F01-MPK007	103.43	14.80^a^	104.70	57.47	6.89	NT	NT	NT
F02-MPN003	95.30	6.30^a^	108.70	105.77	80.74	NT	NT	NT
F03-MPN008	90.52	7.20^a^	106.20	97.62	35.23	NT	NT	NT
F04-MPN007	90.84	7.20^a^	106.87	105.13	105.99	NT	NT	NT
F05-MPK003	103.51	108.80^b^	NT	91.26	40.70	NT	NT	NT
F06-MPK008	105.42	104.00^b^	89.80	104.92	18.96	NT	NT	NT
F07-MPN002	67.81	87.30^b^	73.30	35.65	1.56	NT	NT	NT
F08-MPK010	92.91	NT^c^	5.30	NT	63.40^d^	NT	109.42	98.70
F09- MPK006	89.96	NT	NT	NT	29.70^d^	NT	101.61	109.13
F10- MPK001	99.92	NT	NT	NT	84.60^d^	108.07	NT	100.26

a Serum collection before colostrum receiving,

b Serum collection after colostrum receiving,

c Not test,

d Vaccinated day

## Discussion

The gold standard of AHS control and prevention in epizootic countries comprises several strategies such as movement restrictions, quarantine, vector elimination, and vaccination [11]. Nonetheless, laboratory diagnosis remains essential for demonstrating freedom from AHS infection in equine/horse populations as well as for evaluating the efficiency of eradication policies, confirming clinical cases, estimating the prevalence of AHS, and evaluating the post-vaccination immune status of individual horses or populations [12]. In both endemic and epidemic AHS scenarios [13], including in Africa, live-attenuated AHS vaccination is a strategy to reduce the impact of the disease in endemic regions and eradicate AHS in epidemic areas [14, 15]. However, it is necessary to consider the post-vaccination immune response of individual animals or populations and the side effects.

The present study evaluated the humoral immune response before and after a single vaccination using a live-attenuated AHSV vaccine in stallions, non-pregnant mares, and pregnant mares, including maternal immunity in foals, during the first disease outbreak in Thailand. To the best of our knowledge, this is also the first report in this country. After vaccination in stallions, non-pregnant mares, and pregnant mares, the antibody titer increased above the cutoff positive level (BP > 50) and remained steady for at least 12 months post-vaccination. An analysis of the influencing factors, including gender and stage of gestation, revealed no statistical difference in antibody titers after AHS vaccination in each selected group. The results of this study are comparable with the results reported by Roddriguez *et al*. [16], who evaluated the immune response of horses to inactivated AHS vaccines and showed that antibody titers remained high for 12 months and increased strongly after the annual booster. Furthermore, there were substantial increases in maternal immunity in foals after receiving colostrum, confirming that passive immunity could be transferred through colostrum but not by the placenta, which correlated well with the results reported by Crafford *et al*. [17] and Alexander and Mason [18].

When live-attenuated vaccines are used, it is necessary to consider several concerns, such as their possible reversion to virulence, transmission, genetic reassortment with field AHSV strains, and the inability to differentiate between infected and vaccinated animals [19, 20]. The recovery of AHS-free status from OIE must be demonstrated, such as without AHS infection for at least the past 2 years and no routine AHS vaccination during the past year [11]. Thailand had reported no cases of AHS since September 2020, and AHS vaccination was abandoned in 2021. Therefore, OIE has just approved the AHS-free status to Thailand on March 10, 2023.

## Conclusion

Single AHS vaccination using a live-attenuated vaccine could stimulate antibody titers that are sufficiently high to control and prevent AHS during disease outbreaks in Thailand. After vaccination, the immune response of both genders, including pregnant mares of various gestation stages, was statistically not different.

## Authors’ Contributions

NK, MT, UK, KR, TM, KT, TP, and SR: Study conception and design, conducted the experiments, and analyzed the data. NK and SR: Contributed to sample preparation. NK, TM, and SR: Drafted the manuscript. All authors have read, reviewed, and approved the final manuscript.
